# Shaeer’s hydro-inflation technique for neurovascular bundle mobilization during penile surgery

**DOI:** 10.1038/s41443-025-01153-z

**Published:** 2025-08-21

**Authors:** Osama Shaeer, Hossam El Debs, Amr Elahwany, Kamal O. K. M. Shaeer, Kamal Shaeer

**Affiliations:** 1 Department of Andrology, Kasr El Aini Faculty of Medicine, Cairo University, Cairo, Egypt; 2 Medical School, Kasr El Aini Faculty of Medicine, Cairo University, Cairo, Egypt

**Keywords:** Erectile dysfunction, Sexual dysfunction

## Abstract

Neurovascular bundle mobilization (NVBm) requires experience to avoid injury of the dorsal nerves and arteries of the penis. This work described Shaeer’s Hydro-Inflation Technique for Neurovascular Bundle Mobilization (S-NVBm), whereby infiltration of the neurovascular bundle with saline is performed to increase safety and speed of NVBm. S-NVBm was performed in 50 cases: 21 cases of corporal rotation for congenital curvature, and 29 cases of penile prosthesis implantation with slitting of the tunica albuginea for correction of Peyronie’s disease deformity (S-NVBm group). A matching group was operated upon with “classic” NVBm, without hydro-inflation (C-NVBm group, *n* = 32). In S-NVBm cases, hydro-inflation of Buck’s fascia was performed prior to NVBm using 80% saline and 20% xylocaine (without adrenaline). The mixture was injected into Buck’s fascia with the blunt nozzle of a 20 ml syringe, superficially applied to the surface. Average duration for NVBm in the S-NVBm group was 3.5 min ± 1.4 (range 1.2–7), compared to a duration of 7.3 ± 2 (range 4–11.2) in the C-NVBm group (*p* < 0.001); a 51.8% difference. No arterial injury was witnessed with S-NVBm group, compared to 1 case of minor unilateral arterial injury in the C-NVBm group. Sensitivity score was 10.2% higher in the S-NVBm group (mean 4.7 ± 0.5, range 3–5) compared to a mean of 4.3 ± 1 (range 2–5) in the C-NVBm group (*p* < 0.001). Biosthesiometry detected a mild sensory deficit in 1/50 cases of the S-NVBm group (2%) compared to 3/32 in the C-NVBm group (9.4%). Average post-operative pain score was 46% lower (2.5 ± 1.4, range 1–6) in the S-NVBm group compared to 4.6 ± 1.3 (range 2–7) in the C-NVBm group(*p* < 0.001). The findings herein demonstrate that Hydro-Inflation technique allows mobilization of the neurovascular bundle in a shorter time, with less post-operative pain, and with a lower risk for sensory deficit.

## Introduction

The neurovascular bundle (NVB) is the layer that holds the dual dorsal nerves, the two dorsal arteries, and the deep dorsal vein of the penis, within Buck’s fascia. Along the shaft of the penis, NVB envelops both corpora cavernosa and extends from the 11 to 1 o’clock positions relative to the corpus spongiosum [1]. The main trunks of the dorsal nerves, arteries and vein course on the dorsal aspect, with the deep dorsal vein in the midline [2, 3]. Neurovascular bundle mobilization (NVBm) is a key-step in many reconstructive penile surgeries. Procedures involving NVBm include hypospadias repair, correction of congenital penile curvature, Peyronie’s disease (PD) plaque surgery with or without penile prosthesis implantation (PPI), sliding technique, penile disassembly, among others. In more complex procedures, significant complications have been reported, including glans necrosis [4]. The “ESSM Position Statement on Surgical Treatment of PD” states that following surgery, development of de-novo erectile dysfunction can be a complication of extensive NVBm [5]. Glans hypoesthesia has been reported following surgery for congenital curvature in 4.3–29.8%, and for PD in 7.9–31.3% [6, 7].

**Fig. 1:**
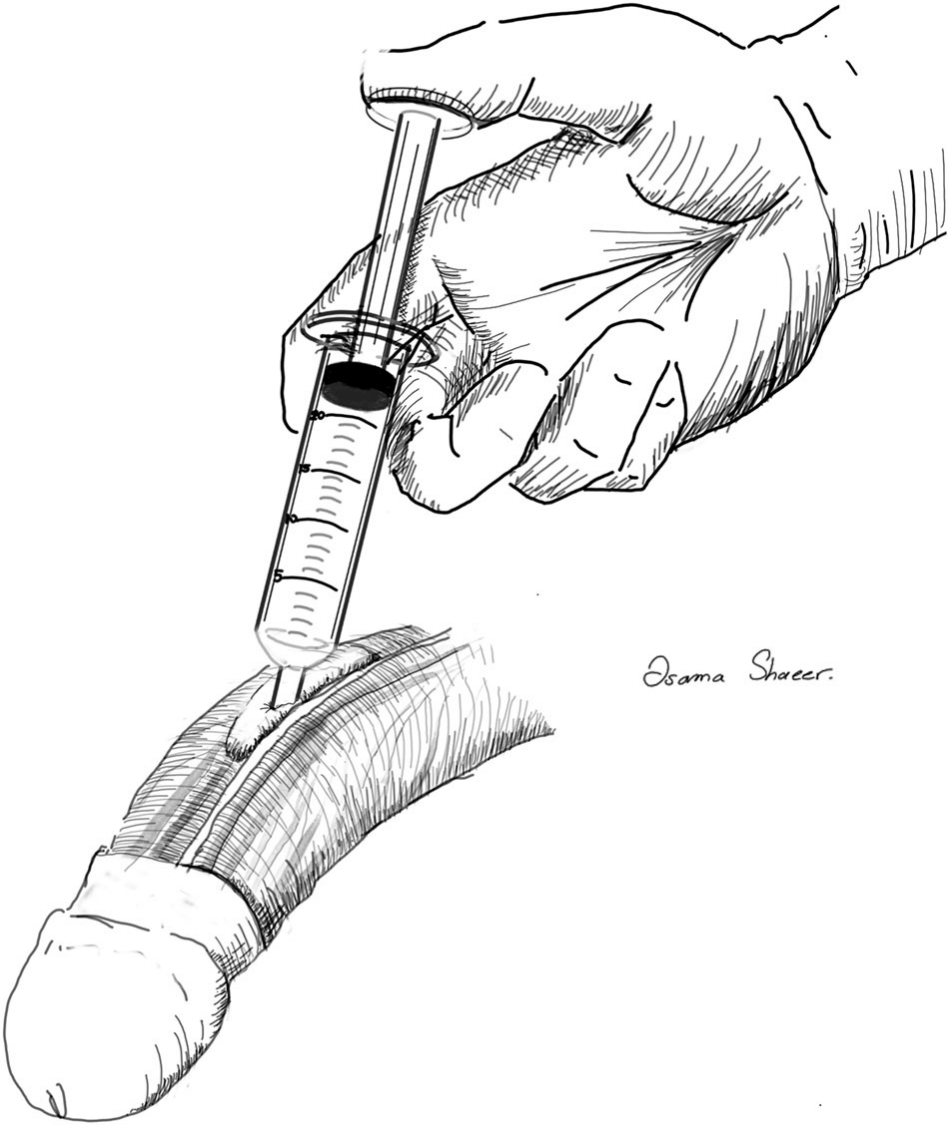
Illustration of hydro-inflation of Buck’s fascia with saline (S-NVBm).

This work described Shaeer’s Hydro-Inflation Technique for NVBm (S-NVBm), whereby hydro-inflation of the NVB is performed to increase safety and speed of mobilization. Buck’s fascia is infiltrated with saline (Fig. 1). This adds tissue volume to the NVB as a safety margin around the nerves and vessels coursing through Buck’s fascia, potentially protecting them during mobilization. Hydro-dissection also widens interstitial planes and increases the distinction between them. This may facilitate tissue separation, rendering dissection of the plane between the tunica albuginea and Buck’s fascia easier and faster.

## Subjects and methods

In a single-center prospective study, S-NVBm was performed in a total of 50 cases: 21 cases of severe ventral congenital penile curvature and 29 cases of PD deformity and refractory erectile dysfunction. Cases with congenital curvature were corrected by Corporal Rotation (CR) [8]. PD cases were managed by PPI, along with the tunica albuginea slitting (PPI-S). Those cases comprised the study group (S-NVBm group, *n* = 50). A matching group was operated upon with “classic” NVBm, without hydro-inflation (C-NVBm group, *n* = 32): 12 cases of CR for severe ventral congenital curvature, and 20 PD cases managed with PPI-S. In both groups, surgery was performed through a subcoronal degloving incision. Patients provided a written informed consent and were randomized into the fore mentioned groups. Ethical approval was obtained from the Department of Andrology, Kasr Al-Aini Faculty of Medicine, Cairo University, Egypt.

### Classic NVBm in the C-NVBm group

In the C-NVBm group, NVBm started with sharp cutting of the Buck’s fascia along the longitudinal axis of the shaft, then undermining and elevating the NVB within Buck’s fascia by alternating sharp and blunt dissection, aided with bipolar diathermy. Ligation of circumflex veins was performed, when needed. We limit NVBm to the minimum needed to perform the procedure, and we try to avoid full NVBm. For PD cases, cutting Buck’s fascia open was performed either at the dorsal midline, or ventrally, adjacent to the spongiosum, according to which is closest to the point of maximum curvature or PD plaque. For CR cases, Bucks’ fascia was cut open dorsally, on either side of the deep dorsal vein.

### Hydro-inflation in the S-NVBm group

**Fig. 2:**
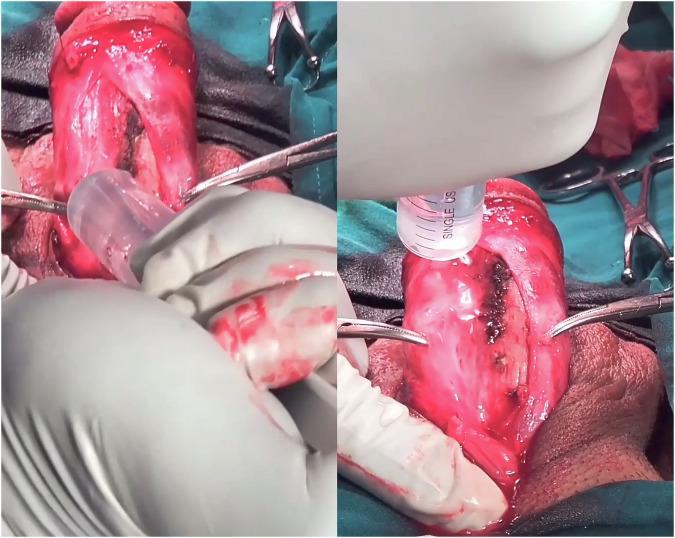
Hydro-inflation.

**Fig. 3:**
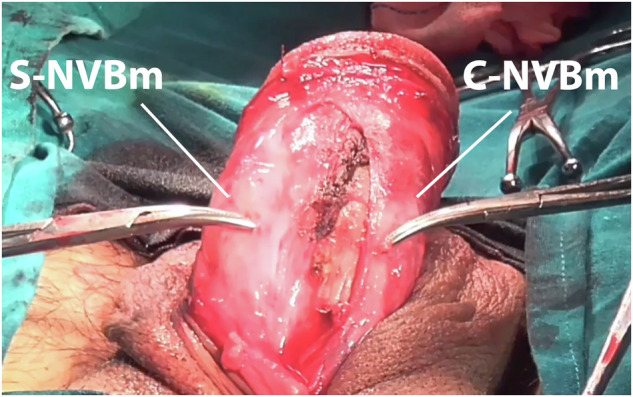
NVB infiltrated with saline upon S-NVBm, compared to C-NVBm.

In S-NVBm cases, Buck’s fascia was cut as fore mentioned with the C-NVBm group. Hydro-inflation of Buck’s fascia was performed prior to mobilization using 80% saline and 20% xylocaine (without adrenaline). The mixture was injected into Buck’s fascia with the blunt nozzle of a 20 ml syringe, superficially applied to the surface (Fig. 2). Face shields were used for protection against the possible spray-back. Injection resulted in bleb formation, and was repeated at several points all over the area intended for mobilization. Following hydro-inflation, mobilization proceeded as with the C-NVBm group: alternating sharp and blunt dissection, aided with bipolar diathermy (Fig. 3). In the rare instances where Buck’s fascia did not take-in the injected fluid, a butterfly cannula was inserted along the longitudinal axis of the shaft (along the NVB structures), and the fluid was infiltrated at the desired points.

The two groups were compared regarding: duration for NVBm, intra-operative injury to the dorsal arteries of the penis, first week post-operative pain, and post-operative sensory deficit at the 6th month. Pain was evaluated on a scale from zero to ten, ten being the most severe. Sensory deficit was evaluated both subjectively (on a 5-point scale, zero being complete loss of sensitivity), and objectively using the biosthesiometer (Bio-medical Instrument CO., Newbury, OH, USA). Biosthesiometry is a non-invasive method that evaluates sensitivity of the penis by comparing perception of vibratory stimuli in the glans, to the thumb. Output is in the form of penile sensitivity ratio (PSR) [9]. Normal PSR was set as 1, where the threshold for perceiving vibratory stimuli is equal in both the tip of the thumb and in the penis. PSR down to 0.75 was considered a mild sensory deficit, 0.5 moderate, and less than 0.5: severe.

All surgeries were performed by a single high-volume surgeon. Video of Shaeer’s Hydro-Inflation Technique for NVBm is available at the Video Journal of Sexual Medicine [10] (www.vjsm.info).

## Results

None of the patients included in this study had previous surgery in the penis, abdomen, pelvis or spine. None of the CR patients were diabetic. In the PPI-S patients, diabetics had strict diabetic control before surgery with HBA1c below 7, and were randomized into the two groups such that their percentage within the two groups was comparable: 45% of PPI-S cases in the S-NVBm group (9/29), and 42.9% in the C-NVBm group (6/20).

Average duration for NVBm in the S-NVBm group was 3.5 min ± 1.4 (range 1.2–7), compared to a duration of 7.3 ± 2 (range 4–11.2) in the C-NVBm group (*p* < 0.001); a 51.8% difference. No arterial injury was witnessed with S-NVBm group, compared to 1 case of minor unilateral arterial injury in the C-NVBm group. Sensitivity score was 10.2% higher in the S-NVBm group (mean 4.7 ± 0.5, range 3–5) compared to a mean of 4.3 ± 1 (range 2–5) in the C-NVBm group (*p* < 0.001). Biosthesiometry detected a mild sensory deficit in 1/50 cases of the S-NVBm group (2%) compared to 3/32 in the C-NVBm group (9.4%). Average post-operative pain score was 46% lower (2.5 ± 1.4, range 1–6) in the S-NVBm group compared to 4.6 ± 1.3 (range 2–7) in the C-NVBm group(*p* < 0.001), (Table 1). Finally, the personal impression of the authors is that NVB with hydro-inflation was much easier and faster than NVB without hydro-inflation.

**Table 1:** Results.

	NVBm Duration (minutes)		Pain Score		Sensitivity Score	
	S-NVBm	C-NVBm	S-NVBm	C-NVBm	S-NVBm	C-NVBm
Mean	3.5	7.3	2.5	4.6	4.7	4.3
St.Dev.	1.4	2.0	1.4	1.3	0.5	1.0
*p* Value	0.000		0.000		0.005	
% Difference	51.8		46.0		−10.2	

	Arterial Injury		Biosthesiometry			
	S-NVBm	C-NVBm	S-NVBm	C-NVBm		
Number	0/50	1/32	1/50	3/32		
%	0	3.1	2	9.4		

*S-NVBm* Shaeer’s hydro-inflation technique for neurovascular bundle mobilization, *C-NVBm* classic neurovascular bundle mobilization.

## Discussion

In penile surgery, every effort should be exerted to preserve the NVB in order to avoid a permanent sensory deficit or devascularization. This is even more crucial in patients with peripheral neuropathy and vasculopathy, such as diabetic patients, who comprise a large sector of patients with erectile dysfunction and PD.

Several variations of NVBm have been invented to enhance its safety [11, 12]. NVBm is classically commenced from the ventral side, proceeding laterally, then dorsally upto the deep dorsal vein (the lateral approach). Alternatively, NVBm can be performed from the dorsal midline, laterally, then ventrally (the medial approach) [11]. The availability of both approaches may help preserve the NVB by selecting the approach closest to the site of intended intervention, and mobilizing the bundle to the least extent that would allow the repair, as opposed to full mobilization. Dean et al. proposed using a 4-mm-wide Freer elevator that is positioned under Buck’s fascia while hugging the tunica albuginea, sliding it until separation of both layers [12]. Optical magnification may increase accuracy and safety of NVBm [11].

Furthermore, techniques to de-bulk PD plaques from within the corpora cavernosa have been developed, avoiding the need for NVBm altogether. Penoscopy / Optical Corporotomy is where the resectoscope is used to debulk PD plaques from within [13]. Punch technique is where debulking of plaques is performed using the vertebral punch forceps from within [14]. The Scratch technique involves shaving off plaques by scalpel from within the corpora cavernosa [15].

This study demonstrated a higher safety profile when mobilizing the NVB with prior hydro-inflation. Hydro-inflation of Buck’s fascia creates a swollen layer around the NVB structures, thereby decreasing the liability for injury. There was a lower incidence of injury to the dorsal arteries of the penis. Dorsal nerves of the penis were largely preserved as demonstrated by post-operative sensitivity scale and Biosthesiometry. Hydro-inflation renders Buck’s fascia more readily separable from the tunica albuginea, thereby decreasing operative time. A faster procedure is always welcome with PPI, as it reduces penile prosthesis infection rates [16].

While expert surgeons may be skilled with NVBm, new entrants to prosthetic and reconstructive penile surgery may find NVBm challenging, and the rates of denervation and/or devascularization may be higher than with experts. Hydro-inflation makes the process easier, faster and safer for this subset of less-experienced surgeons. The added ease and speed may still be appreciated by experts, particularly with PD cases where the inflammatory process renders the NVB more adherent to the tunica albuginea. The addition of an anesthetic to the hydro-inflation solution ameliorates post-operative pain.

Among the limitations of the current study are the limited sample size, the single-center experience, and reliance on subjective data for some outcome parameters such as pain evaluation.

In conclusion, the hydro-inflation technique allows mobilization of the neurovascular bundle in a shorter time, with less post-operative pain and lower liability for neurovascular bundle injury.

## Data Availability

Data are available within the published article.
